# SARS-CoV-2 Seroprevalence and Symptom Onset in Culturally Linked Orthodox Jewish Communities Across Multiple Regions in the United States

**DOI:** 10.1001/jamanetworkopen.2021.2816

**Published:** 2021-03-10

**Authors:** Israel Zyskind, Avi Z. Rosenberg, Jason Zimmerman, Hiam Naiditch, Aaron E. Glatt, Abraham Pinter, Elitza S. Theel, Michael J. Joyner, D. Ashley Hill, Miriam R. Lieberman, Elliot Bigajer, Daniel Stok, Elliot Frank, Jonathan I. Silverberg

**Affiliations:** 1 Department of Pediatrics, NYU Langone Medical Center, New York, New York; 2 Maimonides Medical Center, Brooklyn, New York; 3 Department of Pathology, Johns Hopkins University, Baltimore, Maryland; 4 Department of Medicine, Yale University School of Medicine, New Haven, Connecticut; 5 Department of Medicine, Mount Sinai South Nassau, Oceanside, New York; 6 Icahn School of Medicine at Mount Sinai, New York, New York; 7 Public Health Research Institute, New Jersey Medical School, Rutgers, The State University of New Jersey, Newark, New Jersey; 8 Division of Clinical Microbiology, Department of Laboratory Medicine and Pathology, Mayo Clinic, Rochester, Minnesota; 9 Department of Anesthesiology and Perioperative Medicine, Mayo Clinic, Rochester, Minnesota; 10 ResourcePath, Sterling, Virginia; 11 Department of Dermatology, The State University of New York Downstate Medical Center, Brooklyn; 12 Division of Gastroenterology, Department of Medicine, Brookdale University Hospital and Medical Center, Brooklyn, New York; 13 Memorial Sloan Kettering Cancer Center, New York, New York; 14 Division of Infectious Diseases, Department of Medicine, Jersey Shore University Medical Center, Neptune, New Jersey; 15 Hackensack Meridian School of Medicine, Clifton, New Jersey; 16 Department of Dermatology, George Washington University, Washington, DC

## Abstract

**Importance:**

Data on severe acute respiratory syndrome coronavirus 2 (SARS-CoV-2) seroprevalence in the United States are still emerging.

**Objective:**

To elucidate SARS-CoV-2 seroprevalence and symptom onset in a culturally linked community across 5 states in the United States.

**Design, Setting, and Participants:**

This cross-sectional study included adults (aged ≥18 years) recruited from the orthodox Jewish community across 5 states (California, Connecticut, Michigan, New Jersey, and New York) in 3 geographically distinct areas of the United States between May 13 and July 6, 2020. Participants completed an online survey and underwent SARS-CoV-2 antibody testing.

**Main Outcomes and Measures:**

Seroprevalence and date of symptom onset of SARS-CoV-2.

**Results:**

Overall, 9507 adults (mean [SD] age, 39.6 [15.0] years; 3777 [39.7%] women) completed the SARS-CoV-2 survey, of whom 6665 (70.1%) had immunoglobin G anti–SARS-CoV-2 antibody levels assessed. A high seroprevalence of SARS-CoV-2 antibodies was observed across all communities, with the highest proportion of positive testing observed in New Jersey (1080 of 3323 [32.5%]) and New York (671 of 2196 [30.6%]). Most individuals with a positive SARS-CoV-2 immunoglobin G antibody test reported a date of symptom-onset between March 9 and March 31, 2020 (California: 135 of 154 [87.7%]; Connecticut: 32 of 34 [94.1%]; Michigan: 44 of 50 [88.0%]; New Jersey: 964 of 1168 [82.5%]; New York: 571 of 677 [84.3%]). This start date was coincident with the Jewish festival of Purim, celebrated March 9 to 10, 2020, with extensive intracommunity spread in the weeks following (mean and mode of peak symptom onset, March 20, 2020), occurring in the absence of strong general and culture-specific public health directives.

**Conclusions and Relevance:**

This cross-sectional study of orthodox Jewish adults across the US found that socioculturally bound communities experienced early parallel outbreaks in discrete locations, notably prior to substantive medical and governmental directives. Further research should clarify optimal national, local, community-based, and government policies to prevent outbreaks in social and cultural communities that traditionally gather for holidays, assemblies, and festivals.

## Key Points

**Question:**

Can severe acute respiratory syndrome coronavirus 2 (SARS-CoV-2) outbreaks occur simultaneously across culturally bound minority communities?

**Findings:**

In this cross-sectional study of 9507 ambulatory adults, a near simultaneous surge in coronavirus disease 2019 symptom onset and high seroprevalence in as many as 32.5% community members were found among geographically distinct yet culturally bound religious communities. This surge corresponded to social events surrounding the festival of Purim, prior to widespread recognition of epidemic mitigation strategies.

**Meaning:**

These findings suggest that parallel outbreaks may occur within culturally bound communities during holiday periods, which could be exacerbated in the absence of national, culturally sensitive guidance.

## Teaser

This cross-sectional study elucidates the severe acute respiratory syndrome coronavirus 2 (SARS-CoV-2) seroprevalence and onset of coronavirus disease 2019 (COVID-19) symptoms in a culturally linked orthodox Jewish community across 5 US states.

## Introduction

In December 2019, the outbreak of severe acute respiratory syndrome coronavirus 2 (SARS-CoV-2) was reported in Wuhan, Hubei Province, China. Rapid international spread of this virus led to its classification as a pandemic by the World Health Organization (WHO) on March 11, 2020.^1^ The United States initially reported its first cases of SARS-CoV-2 on January 20, 2020, in Snohomish County, Washington.^2^ A subsequent study suggested that SARS-CoV-2 cases already occurred in the United States in December 2019 with community spread that went undetected prior to established clinical awareness and testing capabilities.^3^ As the virus continued to spread, it became evident that transmission was subject to various factors, including contact patterns, symptomatology, age, and adoption of mitigation measures.^4,5^ Several reports highlighted the importance of the sociocultural and religious context in which such transmission occurred.^6,7,8,9^

In this study, we focus on several geographically distinct but socioculturally interconnected orthodox Jewish communities that experienced dramatic parallel community-based spread following the religious festival of Purim on March 9 to 10, 2020. Around the time of this celebration and in the absence of strong general or culture-specific public health directives, nearly synchronous transmission of infection spread through these distinct and distant, albeit interconnected, communities, ultimately contributing to the significant morbidity and mortality among these communities across multiple states in the ensuing weeks. In this large-scale study, we sought to explore the epidemiology of parallel SARS-CoV-2 outbreaks in a culturally bonded community.

## Methods

### Participant Recruitment

The study design and research protocol were approved by the IntegReview institutional review board. Signed electronic informed consent was obtained from all participants. This study followed the Strengthening the Reporting of Observational Studies in Epidemiology (STROBE) reporting guideline.

The Multi-institutional Study Analyzing Anti–CoV-2 Antibodies (MITZVA) cohort recruited study participants in partnership with local nonprofit and social service organizations offering antibody testing to symptomatic or asymptomatic adults within the large orthodox Jewish communities of Brooklyn, New York; Lakewood, New Jersey; Los Angeles, California; Nassau and Sullivan Counties, New York; New Haven, Connecticut; and Detroit, Michigan. This particular ethnoreligious group tends to live in concentrated geographic areas, is typically close-knit, and holds religion as a central part of their lives, allowing for robust recruitment via religiously affiliated venues.^10^ Participants were recruited via paper and social media advertisements that were distributed by local nonprofits and social service organizations with established networks of orthodox Jewish community members. All members of these organizations self-identified with the Jewish community. Recruitment material provided a website address to enroll in this study. There was no compensation for participation. Participants, after inclusion and exclusion criteria were applied, were given a specific time and location to appear for serological testing.

Study inclusion criteria included being 18 years or older, being a man or a woman, being able to sign informed consent, and for those participating in the synchronous antibody testing, agreeing to release antibody data to the study investigators. Exclusion criteria were those who were not able to complete the survey or did not agree to release their antibody data to the investigators.

### Study Design

**Figure 1.**
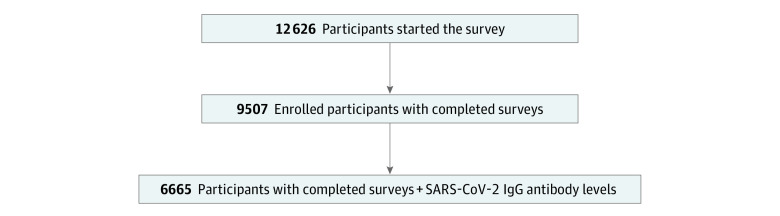
Summary of Study Design IgG indicates immunoglobin G; SARS-CoV-2, severe acute respiratory syndrome coronavirus 2.

This study involved a 2-stage sampling process (Figure 1). Stage 1 was designed to determine the self-reported symptoms and outcomes of SARS-CoV-2 in adults. In this stage, a cross-sectional survey invitation was sent to adults who expressed interest in having antibody testing performed and who were willing to share their SARS-CoV-2 symptom experience. Electronic informed consent and disclosure of the study purpose was performed prior to beginning the survey. A total of 12 626 individuals began the survey process, with a total of 9507 adults completing the survey (completion rate, 75.3%). In stage 2, a subset of 6665 adults (response rate, 70.1%) had antibody testing performed shortly after completing the survey.

### Questionnaire

The questionnaire was developed to determine the most common symptoms and outcomes of SARS-CoV-2. The questionnaire included questions about patient demographic characteristics, whether they had any symptoms of SARS-CoV-2, date of symptom onset, and whether they tested positive for SARS-CoV-2 by nasal swab (eAppendix in the Supplement). The questionnaire was modified based on multiple rounds of feedback from 3 epidemiologists, more than 10 physicians, and 5 local community religious organization partners prior to administration. The questionnaire was then administered via the Health Insurance Portability and Accountability Act–compliant and secure Research Data Capture (REDCap) software.

### Antibody Measurements

Anti–SARS-CoV-2 antibody measurements were performed at the Mayo Clinic Laboratory using the Epitope Diagnostics (EDI) ELISA, established and used for clinical reporting of qualitative test for detection of immunoglobin (Ig) M or G antibodies to the nucleocapsid protein from SARS-CoV-2. For the purposes of this study, IgG results were reported as described previously,^11^ with index value thresholds of 1.21 or greater, 1.01 or less, and between 1.01 and less than 1.21 for positive, negative, and indeterminate results, respectively.^11^ These quantitative indexed measurements are henceforth referred to as antibody levels and are used to compare individual antibody measurements with others in this cohort. All antibody testing was performed between May 14 and 30, 2020.

### Statistical Analysis

All data processing and statistical analyses were performed in SAS version 9.4.3 (SAS Institute). Baseline characteristics were determined, and summary statistics were estimated for the 2 cohorts.

Sensitivity analyses were performed in those who reported a positive SARS-CoV-2 nasal swab and in those who tested positive for SARS-CoV-2 IgG antibodies. The earliest, median, and/or mode dates of symptom onset were determined overall and for each state of residence. The frequency and percentage of SARS-CoV-2 IgG indeterminate and positive levels were determined. Complete data analysis was performed, ie, participants with missing data were excluded. A 2-sided *P* < .05 was considered statistically significant.

## Results

### Population Characteristics

Two distinct cohorts were established in this study. The survey cohort was constituted by 9507 of 12 626 adults who completed the SARS-CoV-2 survey (completion rate, 75.3%). IgG anti–SARS-CoV-2 antibody levels were assessed in a subpopulation of respondents consisting of 6665 adults (70.1% of those who completed the survey), comprising the antibody cohort.

The antibody cohort included individuals residing in 8 distinct communities throughout the US. These include Lakewood, New Jersey (3323 individuals), Brooklyn (Kings County), New York (1298 individuals), Nassau County, New York (754 individuals), Los Angeles, California (684 individuals), Detroit, Michigan (339 individuals), Sullivan County, New York (144 individuals), New Haven, Connecticut (120 individuals) and other (13 individuals). Other included a small subset of individuals residing in Colorado, Florida, Maryland, North Carolina, Ohio, Pennsylvania, and Washington who elected to participate in this study.

The survey cohort was comprised of 3777 women (39.7%) with a mean (SD) age of 39.6 (15.0; range, 18-94 years) from 14 states. The antibody cohort was comprised of 3068 women (46.0%) with a mean (SD) age of 39.6 (14.9) from 12 states. Baseline characteristics for these cohorts are presented in Table 1.

**Table 1.** Population Characteristics

Variable	Subpopulation, No. (%)	
	Survey cohort (N = 9507)^a^	Antibody cohort (n = 6665)^b^
Age, mean (SD) [range]	39.6 (15.0) [18-94]	39.7 (14.9) [18-94]
Sex		
Female	3777 (39.7)	3068 (46.0)
Male	5730 (60.3)	3597 (54.0)
Household size, median (IQR)	5 (3-7)	5 (3-7)
Household sick contact	4870 (60.6)	3636 (61.4)
State of residence		
California	949 (10.0)	684 (10.3)
Connecticut	155 (1.6)	120 (1.8)
Michigan	421 (4.4)	339 (5.1)
New Jersey	4652 (48.9)	3323 (49.9)
New York	3309 (34.8)	2202 (33.0)
Other^c^	20 (0.2)	13 (0.2)
Positive PCR test	603 (6.6)	422 (6.4)

Abbreviations: IQR, interquartile range; PCR, polymerase chain reaction; SARS-CoV-2, severe acute respiratory syndrome coronavirus 2.

^a^ Within the cohort of patients who completed the SARS-CoV-2 survey, missing data were encountered in 1475 participants (15.5%) for age, 1424 (15.0%) for sex, 1 (0.01%) for state of residence, and 319 (3.4%) for PCR testing.

^b^ Within the cohort of patients who completed the SARS-CoV-2 survey and had antibody testing, missing data were encountered in 19 participants (0.3%) for age, 0 for sex, 27 (0.4%) for household size, 743 (11.4%) for household sick contacts, and 27 (0.4%) for PCR testing.

^c^ Other includes people from Colorado, Florida, Maryland, North Carolina, Ohio, Pennsylvania, and Washington.

### Seroprevalence of SARS-CoV-2 Antibodies

High seroprevalence of SARS-CoV-2 antibodies was observed across all communities. The highest proportion of positive or indeterminate testing was observed in New Jersey (positive: 1080 [32.5%]; indeterminate: 190 [5.7%]), followed by New York (positive: 671 of 2196 [30.5%]; indeterminate: 101 [4.6%]), other (positive: 4 of 13 [30.8%]; indeterminate: 0), Connecticut (positive: 35 [29.2%]; indeterminate: 2 [1.7%]), California (positive: 163 [23.8%]; indeterminate: 18 [2.6%]), and Michigan (positive: 51 [15.0%]; indeterminate: 12 [3.5%]) (Table 2).

**Table 2.** Distribution of SARS-CoV-2 IgG Antibody Results by State

State	No.	SARS-CoV-2 IgG antibody test results								
		Negative (n = 4354)			Indeterminate (n = 323)			Positive (n = 2004)		
		No.	% (95% CI)	Seroprevalence, % (95% CI)	No.	% (95% CI)	Seroprevalence, % (95% CI)	No.	% (95% CI)	Seroprevalence, % (95% CI)
California	684	503	7.5 (6.9-8.2)	73.5 (70.2-76.8)	18	0.3 (0.1-0.4)	2.6 (1.4-3.8)	163	2.4 (2.1-2.8)	23.8 (20.6-27.0)
Connecticut	120	83	1.2 (1.0-1.5)	69.2 (60.9-77.4)	2	0.03 (0.0-0.07)	1.7 (0.0-4.0)	35	0.5 (0.4-0.7)	29.2 (21.0-37.3)
Michigan	339	276	4.1 (3.7-4.6)	81.4 (77.3-85.6)	12	0.2 (0.08-0.3)	3.5 (1.6-5.5)	51	0.8 (0.6-1.0)	15.0 (11.2-18.9)
New Jersey	3323	2053	30.7 (29.6-31.8)	61.8 (60.1-63.4)	190	2.8 (2.4-3.2)	5.7 (4.9-6.5)	1080	16.2 (15.3-17.0)	32.5 (30.9-34.1)
New York	2202	1430	21.4 (20.4-22.4)	64.9 (62.9-66.9)	101	1.5 (1.2-1.8)	4.6 (3.7-5.5)	671	10.0 (9.3-10.8)	30.5 (28.5-32.4)
Other^a^	13	9	0.1 (0.05-0.2)	69.2 (44.1-94.3)	0	0.0	0.0	4	0.06 (0.001-0.1)	30.8 (5.7-55.9)

Abbreviations: IgG, immunoglobin G; SARS-CoV-2, severe acute respiratory syndrome coronavirus 2.

^a^ Other includes 13 people from Colorado, Florida, Maryland, North Carolina, Ohio, Pennsylvania, and Washington.

### Date of SARS-CoV-2 Symptom Onset

#### Survey Cohort

**Figure 2.**
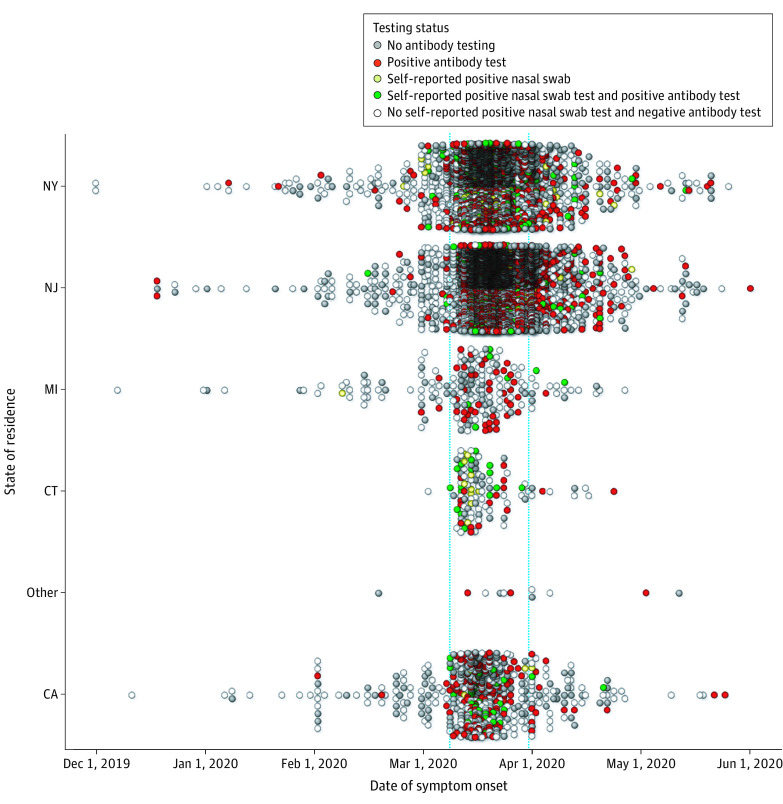
Date of Symptom Onset, by Location and Testing Status Self-reported date of severe acute respiratory syndrome coronavirus 2 symptom onset is presented for each state of residence (CA, CT, MI, NJ, NY, and other, which includes 13 people from Colorado, Florida, Maryland, North Carolina, Ohio, Pennsylvania and Washington). Results are presented for the entire survey cohort. The vertical blue lines indicate the period between March 9 and 31, 2020. All antibody testing was performed between May 14 and 30, 2020.

Across the entire cohort, the date range of possible SARS-CoV-2 symptom onset ranged from December 1, 2019, to May 26, 2020 (median and mode date, March 20, 2020) (Figure 2). The earliest reported date of symptom onset among respondents with a self-reported positive SARS-CoV-2 nasal swab test was on February 8, 2020, in Michigan. Across other states, earliest positive SARS-CoV-2 nasal swab tests were February 15 in New Jersey, February 25 in New York, and March 9 in California and Connecticut. The median and mode dates of symptom onset occurred within the same 1-week period across all states (California: median and mode, March 17; Connecticut: median, March 13; mode, March 14; Michigan: median and mode, March 20; New Jersey: median, March 20; mode, March 18; New York: median, March 18; mode, March 17).

Only 415 individuals in the survey cohort (7.2%) reported symptom onset prior to March 9; 4507 (77.7%) reported symptom onset between March 9 and April 1, and 881 (15.2%) reported symptom onset after April 1. Furthermore, most individuals with a positive nasal swab reported their date of symptom onset between March 9 and 31, 2020 (California: 42 of 45 [93.3%]; Connecticut: 32 of 32 [100.0%]; Michigan: 5 of 8 [62.5%]; New Jersey: 174 of 219 [81.7%]; New York: 219 of 268 [81.7%]).

#### Antibody Cohort

Among respondents who tested positive for SARS-CoV-2 IgG antibodies, the earliest dates of COVID-19 symptoms occurred as early as December 18, 2019, in New Jersey; January 7, 2020, in New York; February 1 in California; March 1 in Michigan; and March 9 in Connecticut. Most individuals with a positive SARS-CoV-2 IgG antibody test reported the date of symptom onset between March 9 and March 31, 2020 (California: 135 of 154 [87.7%]; Connecticut: 32 of 34 [94.1%]; Michigan: 44 of 50 [88.0%]; New Jersey: 964 of 1168 [82.5%]; New York: 571 of 677 [84.3%]).

## Discussion

In this large-scale observational MITZVA cohort, we identified parallel SARS-CoV-2 outbreaks in an interconnected sociocultural community across multiple US cities and states. We observed a peak in self-reported symptom onset across 5 states in 3 geographically distinct areas that occurred around the Jewish festival of Purim, a holiday typically observed by gathering to hear the reading of a scroll called a *Megillah*, performing the *mitzva* (good deed) of giving charity to the poor, and partaking in social events, such as religiously mandated holiday feasts and gift-giving. In 2020, Purim was celebrated on March 9 and 10, approximately 7 to 10 days before the reported peak in symptom onset across these culturally bound communities. Of note, the median and mode dates for all of the studied communities ranged in the narrow 4-day interval from March 17 to 21, supporting our hypothesis of parallel spread.

Importantly, the seroprevalance rates recorded through our study demonstrated higher rates than those in neighboring communities, most of which were ethnoculturally distinct. For example, the seroprevalence in the Brooklyn MITZVA cohort was 30.5%, which is markedly higher than the seroprevalence of New York City (16.5%) during the same time period^12^ (Table 2). Similarly, the seroprevalence in our Connecticut MITZVA cohort (29.2%) was much higher than previously reported for Connecticut (4.9%) during the same testing period.^13^

Participants at shared sociocultural and religious events can be particularly susceptible to widespread contagion and its rapid spread, as has been seen previously.^7,14^ Religious and social behaviors are highly interconnected and typically share a symbiotic relationship.^15^ While social gatherings of any kind may be susceptible to outbreaks, the social and communal obligations associated with being in a religious community make the adoption of mitigation strategies, particularly social distancing, a unique challenge.^14,16,17^ As with similar religions with recent festivals, such as the Islamic Hajj, culturally sensitive and clear guidance issued early in a pandemic has the potential to reduce the spread of contagion.^8,16^

The risk to these culturally bound communities was further amplified during the early phase of the pandemic, prior to widespread adoption of social distancing or mask-wearing measures. National directives from public health organizations were still being developed at the time when they would have been most impactful. For example, the WHO first characterized SARS-CoV-2 as a pandemic on March 11,^18^ which was coincidentally the day after Purim. Restrictions on mass gatherings began in mid-March.^19^ Even before such measures were promulgated by public health authorities, local and national orthodox Jewish leadership organizations, with the guidance of public health and medical experts, put forth joint community mandates to prevent the spread of illness. In orthodox Jewish communities across the United States, rabbinic mandates developed culturally sensitive policies to address unique aspects of prayer services, family and communal gatherings, and social support systems, and indeed, many communities closed all religious services and gatherings after Purim—well before local, state, and governmental public health agencies recommended such closures. Indeed, the major orthodox rabbinical authorities from all branches of orthodox Judaism jointly released numerous public declarations in March and April to heed and comply with mitigatory health policies, which resulted in widespread compliance.

Interestingly, multiple SARS-CoV-2 cases were identified with much earlier-than-anticipated dates of symptom onset, including December 2019 and early January 2020. These cases appear to have preceded the first reported case of SARS-CoV-2 in the United States on January 20, 2020.^2^ These results suggest that community transmission of SARS-CoV-2 occurred earlier than previously recognized. A recent study observed a significantly higher number of patients with respiratory complaints and diseases starting in late December 2019 and continuing through February 2020, which may have been secondary to community spread of SARS-CoV-2 prior to established clinical awareness and testing capabilities.^3^ Of note, few patients with positive SARS-CoV-2 antibodies underwent polymerase chain reaction testing, owing to inadequate testing at the time. Thus, SARS-CoV-2 antibody testing may help to provide insight into the level of community exposure to the virus.

We observed a higher level of seroprevalence in these culturally bound communities than previously reported in other studies of the general statewide population in the same areas (eg, 6.9% from March to April in New York).^7,13,20^ Of note, caution should be exercised when comparing these results with trends from some regional surveillance systems in early 2020 that showed very high seroprevalence because these surveys did not perform population or community-based sampling. At that time, there was limited utilization of antibody testing outside of a hospital setting and patients with very severe illness, which would lead to falsely elevated estimates of seroprevalence.

The observed differences in seroprevalence suggest that socioculturally bound communities are more likely to experience widespread community transmission. These results are likely generalizable to many other religious and secular holidays observed in the United States, including Halloween, Thanksgiving, Christmas, and New Year’s. As such, heightened precautions are indicated to avoid similar parallel outbreaks around the country.

To our knowledge, this study represents the largest observational study undertaken in a demographic group about which few observational studies have been previously published. In the wake of an epidemic that ignored religious affiliation, community members exhibited remarkable willingness to collectively participate in an effort to combat coronavirus disease 2019, be it through participating in research projects like our own or enrolling in convalescent serum donation programs. Our hope is that the efforts that went into the facilitation of this study by these thousands of participants will provide the framework for future medical-religious partnerships.

### Limitations

This study has limitations. Despite the large sample size and investigation of multiple geographically diverse communities and regions, our sample population comprised an ambulatory cohort, whose presentation may not accurately reflect the symptomatology and serologic profiles of patients with more severe disease. Ascertainment bias in the surveyed cohort may have additionally led to an overestimation of the seroprevalence of the surveyed population. However, the study was open to all participants and did not require participants to have SARS-CoV-2 symptoms or exposures to participate. Viral polymerase chain reaction positivity was assessed via survey rather than direct testing. The survey was modified based on feedback from multiple stakeholders, although it was not formally pilot tested. Data on travel history and contact tracing were not available. This was a largely Ashkenazi Jewish population and thus almost exclusively Caucasian with limited racial diversity. Future studies are needed to address these limitations.

## Conclusions

In this cross-sectional study of orthodox Jewish adults across the US, culturally bound communities experienced parallel outbreaks of SARS-CoV-2 in geographically discrete locations during the first wave of the coronavirus disease 2019 pandemic in the United States, prior to the widespread recognition and implementation of epidemic mitigation strategies. Future studies should examine the impact of early national, local, and community-driven culturally sensitive policies in religious and socioculturally bound communities.

## Notes

Supplement. **eAppendix.** SARS-CoV-2 Survey
