# Supplementary material for: SARS-CoV-2 Seroprevalence and Symptom Onset in Culturally Linked Orthodox Jewish Communities Across Multiple Regions in the United States
Source: JAMA Netw Open. 2021 Mar 10;4(3):e212816. doi: 10.1001/jamanetworkopen.2021.2816 (PMC7948060; doi:10.1001/jamanetworkopen.2021.2816)
Supplement: Supplement. — eAppendix. SARS-CoV-2 Survey [file jamanetwopen-e212816-s001.pdf]

Supplemental Online Content

Zyskind I, Rosenberg AZ, Zimmerman J, et al. SARS-CoV-2 seroprevalence and symptom onset in culturally linked Orthodox Jewish communities across multiple regions in the United States. *JAMA Netw Open*. 2021;4(3):e212816. doi:10.1001/jamanetworkopen.2021.2816

eAppendix. SARS-CoV-2 Survey

This supplemental material has been provided by the authors to give readers additional information about their work.

© 2021 Zyskind I et al. *JAMA Network Open.*

eAppendix. SARS-CoV-2 Survey

What is your date of birth?	______________________
What is your gender?	◯ Male<br>◯ Female
In what ZIP code is your home located?	______________________
Did you ever have any symptoms or suspicion of having coronavirus?	◯ Yes<br>◯ No
When did your symptoms for Coronavirus begin?<br>Notes for reference:<br>Purim was March 10th<br>Pesach was April 9th	______________________
Did you ever test positive for Coronavirus by nasal swab?	◯ Yes No
How many people live in the same house as you?	______________________
Did any of the people you live with in your house have symptoms or were positive for COVID-19?	◯ Yes ◯ No

© 2021 Zyskind I et al. *JAMA Network Open.*
